# Anti-inflammatory effects of *Stephania tetrandra* S. Moore on interleukin-6 production and experimental inflammatory disease models

**DOI:** 10.1155/S0962935196000415

**Published:** 1996-09

**Authors:** H-S. Kang, Y-H. Kim, C-S. Lee, J-J. Lee, I. Choi, K-H. Pyun

**Affiliations:** 1 Department of Pathology School of Medicine Chungnam University Taejon Korea; 2 Immune Cell Signal Transduction R.U. Korea Research Institute of Bioscience and Biotechnology KIST Yusong, P.O. Box 115 Taejon 305-600 Korea

## Abstract

Deregulation of interleukin-6 (IL-6) expression caused the synthesis and release of many inflammatory mediators. It is involved in chronic inflammation, autoimmune diseases, and malignancy. *Stephania tetrandra* S. Moore is a Chinese medicinal herb which has been used traditionary as a remedy for neuralgia and arthritis in China. To investigate the anti-inflammatory effects of *S. tetrandra* S. Moore *in vitro* and *in vivo*, its effects on the production of IL-6 and inflammatory mediators were analysed. When human monocytes/macrophages stimulated with silica were treated with 0.1–10 μg/ml *S. tetranda* S. Moore, the production of IL-6 was inhibited up to 50%. At these concentrations, it had no cytotoxicity effect on these cells. It also suppressed the production of IL-6 by alveolar macrophages stimulated with silica. In addition, it inhibited the release of superoxide anion and hydrogen peroxide from human monocytes/macrophages. To assess the anti-fibrosis effects of *S. tetrandra* S. Moore, its effects on *in vivo* experimental inflammatory models were evaluated. In the experimental silicosis model, IL-6 activities in the sera and in the culture supernatants of pulmonary fibroblasts were also inhibited by it. *In vitro* and *in vivo* treatment of *S. tetrandra* S. Moore reduced collagen production by rat lung fibroblasts and lung tissue. Also, *S. tetrandra* S. Moore reduced the levels of serum GOT and GPT in the rat cirrhosis model induced by CCL_4_, and it was effective in reducing hepatic fibrosis and nodular formation. Taken together, these data indicate that it has a potent anti-inflammatory and antifibrosis effect by reducing IL-6 production.


Mediators of Inflammation 5, 280–291 (1996)

### Anti-inflammatory effects of *Stephania tetrandra* S. Moore on interleukin-6 production and experimental inflammatory disease models

H-S. Kang, Y-H. Kim, C-S. Lee,[1] J-J. Lee, I. Choi[CA] and K-H. Pyun

Immune Cell Signal Transduction R.U., Korea Research Institute of Bioscience and Biotechnology, KIST, Yusong, P.O. Box 115, Taejon 305-600, Republic of Korea;
[1]Department of Pathology, School of Medicine, Chungnam University, Taejon, Republic of Korea

[CA]Corresponding Author

**Deregulation of interleukin-6 (IL-6) expression caused the synthesis and release of many inflammatory mediators. It is involved in chronic inflammation, autoimmune diseases, and malignancy. *Stephania tetrandra* S. Moore is a Chinese medicinal herb which has been used traditionally as a remedy for neuralgia and arthritis in China. To investigate the anti-inflammatory effects of *S. tetrandra* S. Moore *in vitro* and *in vivo*, its effects on the production of IL-6 and inflammatory mediators were analysed. When human monocytes/macrophages stimulated with silica were treated with 0.1–10 μg/ml *S. tetranda* S. Moore, the production of IL-6 was inhibited up to 50%. At these concentrations, it had no cytotoxicity effect on these cells. It also suppressed the production of IL-6 by alveolar macrophages stimulated with silica. In addition, it inhibited the release of superoxide anion and hydrogen peroxide from human monocytes/macrophages. To assess the anti-fibrosis effects of *S. tetrandra* S. Moore, its effects on *in vivo* experimental inflammatory models were evaluated. In the experimental silicosis model, IL-6 activities in the sera and in the culture supernatants of pulmonary fibroblasts were also inhibited by it. *In vitro* and *in vivo* treatment of *S. tetrandra* S. Moore reduced collagen production by rat lung fibroblasts and lung tissue. Also, *S. tetrandra* S. Moore reduced the levels of serum GOT and GPT in the rat cirrhosis model induced by $CCl_4$, and it was effective in reducing hepatic fibrosis and nodular formation. Taken together, these data indicate that it has a potent anti-inflammatory and anti-fibrosis effect by reducing IL-6 production.**

**Key words:** Anti-fibrosis, Anti-inflammation, IL-6 production, *Stephania tetrandra*

### Introduction

Inflammation is initiated by the infiltration of inflammatory, immune and mesenchymal cells into the inflamed sites. Monocytes/macrophages involved in the influx of these cells into the inflamed site produce soluble mediators.[1] Once these cells have entered the inflammatory sites, they begin to proliferate and initiate the synthesizing of inflammatory and fibrogenic factors such as collagen. The repair of tissue injury results in replacement of the original tissue by collagen. This collagen production not only serves to repair the damaged tissue, but may also cause damage to the organ.[2–4]

Silicosis is an occupational lung disease resulting from the inhalation of silica dust which causes chronic inflammation and progressive pulmonary fibrosis. Inhaled silica particles are first ingested by alveolar monocytes/macrophages, which then release oxygen free radicals and lysosomal enzymes which damage lung tissue.[5] They also release pro-inflammatory cytokines which recruit more inflammatory cells to secrete these fibrogenic mediators inducing the proliferation of fibroblasts and collagen synthesis.[6,7] Hepatic fibrosis is one of the chronic inflammatory liver diseases. It is a complex process that involves the deposition of extracellular matrix components, activation of cells capable of producing inflammatory mediators, cytokines, and tissue remodelling. Unbalanced production of pro-inflammatory cytokines and inflammatory mediators has been reported in chronic liver diseases.[8,9] In cirrhotic liver, levels of IL-1, TNF, and IL-6 are increased. TGF-β is also involved in collagen synthesis and chronic liver diseases.

As is well known, IL-6 is a regulatory factor

© 1996 Rapid Science Publishers which participates in the growth, differentiation and activation of cells. It is produced and secreted by various organ cells, and plays an important role in defensive mechanisms of the human body.[10] IL-6, first discovered in the culture supernatants of monocytes, has been reported to induce the production of antibodies by B cells.[11] Since the successful cloning of the cDNA of IL-6,[12] IL-6 has been reported to serve as a growth factor for B cell hybridoma and plasmacytoma,[13] and as a factor participating in haematopoiesis.[14] Further, IL-6 has been reported to have the functions of stimulating the activation and growth of T cells;[15] inducing the acute phase response of liver cells;[16] regulating cell differentiation in the nerve system;[17] stimulating the growth of keratinocytes; regulating bone metabolism; stimulating the growth of kidney mesangial cells; and inhibiting the growth of melanoma and breast cancer cells, etc. As has been reported, various diseases may be the results of the improper regulation of IL-6 production. Examples of the diseases reported are rheumatoid arthritis,[18] hepatocirrhosis,[19] psoriasis,[20] multiple myeloma,[21] cardiac myxoma,[22] AIDS[23] and other autoimmune diseases. These observations have buttressed the importance of regulating IL-6 production for the maintenance of the homeostasis of the immune system in the human body and for the treatment and prophylaxis of diseases.

Numerous approaches have been proposed to regulate the production of interleukins. For instance, proliferation of myelocytes in a patient suffering from myeloma caused by an excessive secretion of IL-6 has been suppressed by employing antibodies against IL-6 or IL-6 receptor.[24] However, no substance or method has been reported to inhibit specifically the production of IL-6 and, therefore, there has still existed a need for the discovery of specific inhibitors against the production of IL-6.

*S. japonica* Miers and *Sinomenium acutum* Rehd et Wils (Menispermaceae), which are found in the southern part of the Republic of Korea, have been used for a long time as an analgesic and anti-inflammatory agent. On the other hand, *S. tetrandra* S. Moore (Menispermaceae), which is not found in the Republic of Korea, has been used traditionally as remedies for neuralgia and arthritis, e.g. in China. Especially, the alkaloid tetrandrine has been used as an anti-inflammatory and anti-hypertensive agent. *S. tetrandra* S. Moore has been reported to have anti-phagocytic and anti-oxidizing effects, and to exhibit effectiveness in clinical and experimental silicosis models[25,26] and is known to have the ability to inhibit the production of interleukin-1 and tumour necrosis factor-α which are secreted by human monocytes.[27,28] Tetrandrine and its derivatives are reported to promote brain function and have been developed as an antimalarial drug and also a stimulant for hair growth.

In this study, to investigate the potent anti-inflammatory inhibitors, extracts from the root of *S. tetrandra* S. Moore were tested to suppress the production of IL-6 and inflammatory mediators. It successfully inhibited IL-6 production, and showed anti-inflammatory, anti-silicosis, and anti-cirrhotic effects *in vivo* and *in vitro.*

### Materials and methods

*Isolation of extracts of* S. tetrandra *S. Moore:* About 4.0 kg of well dried root of *S. tetrandra* S. Moore was chopped and extracted with about 5 l of methanol for 2 days. The extraction procedure was repeated three times and the combined extracts were concentrated under a reduced pressure to obtain about 224 g of the methanol extract (Extract A) with a yield of 5.6%. Two hundred grams of Extract A was partitioned with 500 ml of 90% methanol and 500 ml of *n*-hexane. The 90% methanol layer was separated and concentrated under reduced pressure to remove methanol. The residue was adjusted to pH 10 with 0.1 M $NH_4OH$ and partitioned with 600 ml of distilled water: $CH_2Cl_2$(1:1(v/v)) mixture. The $CH_2Cl_2$ layer, i.e. the alkaloid fraction was then separated and concentrated under a reduced pressure to obtain about 25 g of Extract B with a yield of 0.6%. Extract C for use in a test for the treatment of silicosis was prepared as follows. One kilogram of dried root of *S. tetrandra* S. Moore was crushed into powder, sieved (60 mesh) and then suspended in distilled water in a concentration of 100 mg/ml. The resulting suspension was heated at 100°C for 6 h and filtered. The filtrate was concentrated under reduced pressure to obtain 80 g of water extract of *S. tetrandra* S. Moore (Extract C) with a yield of 8%, which was then stored at –20°C. For the purpose of preparing Extract C for the treatment of hepatocirrhosis, 1113.5 g of dried root of *S. tetrandra* S. Moore was introduced in a 3 l round-bottomed flask equipped with a cooling apparatus with 2 l of distilled water, and the mixture was heated at 95°C for 12 h and then filtered. The filtrate was concentrated under a reduced pressure by employing a rotary vacuum evaporator (Buchi 451), frozen in a deepfreeze (Sanyo, Japan) at –84°C for 3 h, and then lyophilized for 4 h by employing a lyophilizer (Eyela, Japan) to obtain 56.55 g of powdery Extract C with a yield of 5.1%. Further, 500 g of dried root of *S. tetrandra* S. Moore was extracted with about 1.5 l of ethanol at room temperature for 3 days. The extraction procedure was repeated three times and the combined extracts were concentrated under reduced pressure to obtain 13 g of ethanol extract of *S. tetrandra* S. Moore with a yield of 2.6% (Extract D).

*Cell culture:*

(1) *Separation of human monocytes/macrophages.* Heparinized normal human peripheral blood was diluted with an equal amount of Hank's balanced salt solution (HBSS: $Ca^{2+}$ and $Mg^{2+}$ free). The diluted blood was put into a centrifuge tube containing two layers of Ficoll-Hypaque (Sigma, St Louis, MO, USA), one with a density of 1.077 and other with a density of 1.119, and then centrifuged at 700 × **g** for 30 min to obtain monocytes from the layer between Ficoll-Hypaque layer with a density of 1.077 and serum layer. The separated cells were washed twice with 4°C HBSS ($Ca^{2+}$ and $Mg^{2+}$ free) and resuspended in RPMI 1640 medium (Gibco, Grand Island, NY, USA) containing 10% foetal bovine serum (FBS, Hyclone, Logan, UT, USA). The suspensions were added to the wells of a 24-well incubation plate (Costar, Cambridge, MA, USA) and incubated at 37°C for 2 h to obtain monocytes/macrophages.

(2) *Separation of alveolar macrophages.* Rat alveolar macrophages were obtained by bronchoalveolar lavage.[29] Rats were anaesthetized with ketamine and their alveolar macrophage were obtained therefrom by inserting a sterilized thin tube into the bronchia and repeating three times the injection of 10 ml of RPMI 1640 medium with a 30 ml syringe. The obtained cells were centrifuged at 400 × **g** for 5 min, suspended in 50 ml of RPMI 1640 medium containing 10% FBS and then incubated at 37°C for 2 h to adhere to the incubation plate. The plate was washed twice with washing buffer (PBS) to remove alveolar lymphocytes (floating cells) and to obtain alveolar macrophages.

The alveolar macrophages (2 × $10^5$ cells/well) were added to the wells of a 24-well incubation plate and treated with 100 μg/ml of silica and 10 μg/ml of Extract A for 3 days. The culture was centrifuged to obtain the culture supernatants, which were then dialyzed against PBS. The activity of IL-6 was determined using an IL-6 dependent B9.55 hybridoma cell line.

(3) *Separation of synoviocytes from patients with rheumatoid arthritis.* Synovial membrane tissue of a patient suffering from rheumatoid arthritis was washed three times with cool PBS and cut into small pieces, which was then suspended in Dulbecco's Modified Eagle's Medium (DMEM; Sigma, USA) containing collagenase A (5 mg/ml, BM, Indianapolis, IN, USA) and DNase type I (0.15 mg/ml, Sigma), and incubated at 37°C for 2 h in a 5% $CO_2$ incubator. Then, 0.5% trypsin-0.2% EDTA was added and the incubation was continued for 30 min. The digested tissue was washed twice with PBS and once with DMEM, and isolated cells were suspended in DMEM containing 10% FBS (DMEM-10% FBS) and incubated for 1 week. Thereafter, synovial adherent cells were isolated with trypsin-EDTA, washed with DMEM and then suspended in DMEM-5% FBS in a concentration of $10^5$ cells/ml.

(4) *Treatment of cells with extracts of* S. tetrandra *S. Moore.* Extracts of *S. tetrandra* S. Moore were added in various concentrations to 1–5 × $10^5$/ml of cells which were obtained in the above procedures, and the cells were preincubated at 37°C for 1 h in 5% $CO_2$ incubator. Then, 1 ml each of silica (100 μg/ml) and RPMI 1640 medium containing 2% FBS were added and the cells were cultured under the same conditions as above for 48 h. The culture supernatant was collected and centrifuged at 1 500 rpm for 10 min to remove the cells and silica. The obtained supernatant was dialyzed against PBS and filtered by 0.2 μm filtration syringe, and the filtrate was stored at –20°C.

*Assay for cytotoxicities of Extracts of* S. tetrandra *S. Moore.* The cytotoxicities of the extracts of *S. tetrandra* S. Moore were determined by the following procedures. Briefly, 5 × $10^5$ cells/ml each of monocytes and macrophages were treated with 0.1, 1 and 10 μg/ml each of Extracts A and B obtained as described above and incubated under the same conditions. In accordance with the method of Alley *et al.*,[30] the culture was added to the wells of the incubation plate, and 100 μg of 3–4,5-dimethylthiazol-2,5-diphenyl-tetrazolium bromide (MTT, Sigma) was added to each of the wells. After incubating at 37°C for 4 h, the culture was centrifuged to remove supernatant. One hundred each of acidified isopropanol (0.04 N HCl in isopropanol) was added to the cells in each well to elute formazan produced by the living cells, and optical density (O.D.) was determined at 540 nm by using an ELISA reader (Titertek multiskan Mcc/340).

*Cytokine assay:* TNF-α activity was measured by cytolysis of TNF-α sensitive murine fibroblast cell line, L929 cells (ATCC, CCL 1, NCTC clone 929). In brief, 3 × $10^4$ L929 cells were plated on a 96-well tissue culture plate. After 24 h incubation, media were removed and various dilutions of samples or various concentrations of recombinant human TNF-α (Genzyme, Cambridge, MA; specific activity 2 × $10^7$ U/mg) were added in duplicate to each well containing 1 µg/ml (final concentration) of actinomycin D (Sigma) and 5% FBS. After 18 h incubation at 37°C, media were removed and cells were washed three times and stained with 0.5% crystal violet solution containing 20% methanol. After washing off the excess dye, the plates were dried and 33% (v/v) acetic acid was added to each well (100 µl/well) and the optical density at 570 nm was measured with an ELISA reader. TNF activity was expressed as U/ml from the extrapolation of the standard curve obtained using recombinant human TNF-α (Genzyme). The activity of IL-6 was determined by using an IL-6 dependent B9.55 hybridoma cell line. B9.55 cells were cultured in RPMI 1640 medium containing 10% FBS with the addition of 2 U/ml of recombinant human IL-6, and the cells were washed three times with serum-free medium. The cells were suspended in RPMI 1640 medium containing 10% FBS in a concentration of 5 × $10^4$ cells/ml, and the suspension was added to the wells of a 96-well incubation plate in an amount of 100 µl/well. Then the plate was incubated at 37°C under 5% $CO_2$ for 68 h. [$^3$H]thymidine (0.5 µCi) was added to the wells in the amount of 50 µl/well and incubation was continued for 4 h. When the incubation was completed, the cells were collected on a glass fibre filter by using a multiple cell harvester (Inotech) and the amount of incorporated [$^3$H]thymidine was determined by a liquid scintillation counter (Beckman, Somerset, NJ, USA). IL-6 ELISA was performed using IL-6-specific monoclonal and polyclonal antibodies obtained from Genzyme (Cambridge, MA). IL-1 activity was determined using thymocyte costimulation assay as described. Briefly, thymocytes were prepared from female $C_3H$/HeJ mice and resuspended in RPMI containing 10% FBS and 2 µg/ml phytohaemagglutinin. 1 × $10^6$ cells (100 µl) were plated on a 96-well tissue culture plate and various dilutions of samples (100 µl) were added to each well. After 44 h incubation, cells were labelled with 0.5 µCi/well [$^3$H]thymidine for 4 h. One unit of cytokine was defined as the amount inducing the half maximal proliferation.

*IL-6 gene expression:* The synoviocytes (1.5 × $10^6$ cells/well) were incubated at 37°C under 5% $CO_2$ for 24 h until it adhered to the wells. One µg/ml or 10 µg/ml of Extract B was added to the wells and the plate was incubated at 37°C under 5% $CO_2$ for 1 h and stimulated with 100 µg/ml silica for 12 h. Then, the culture medium was removed, and the cells were washed with PBS, disrupted by adding 500 µl of denaturating solution (4 M guanidinium thiocyanate, 25 mM sodium citrate, pH 7.0, 0.1 M 2-mercapto-ethanol, 0.5% sarcosine) to obtain RNA. To synthesize a single-strand cDNA from the RNA obtained above, a reverse transcription reaction was carried out by employing M-MLV reverse transcriptase (Promega, USA). Then, a polymerase chain reaction (PCR) was carried out to amplify cDNA. Twenty µl of reverse transcription reaction, 8 µl of 10 × PCR buffer (100 mM Tris-HCl, pH 8.3, 400 mM KCl, 120 mM DTT, 15 mM $MgCl_2$, 5 µg/ml BSA), 1 µl (20 pmol) of 5′-end primer (5′-ATGAACTCCTTCTCCACAAGCGC-3′), 1 µl (20 pmol) of 3′-end primer (5′-GAAGAG-CCCTCAGGCTGG-ACTG-3′), 69 µl of distilled water and 1 µl (2.5 U) of Taq DNA polymerase (Promega, USA) were mixed well and the mixture was incubated at 95°C for 5 min. The PCR was carried out by repeating 30 times the thermal cycle consisting of 95°C for 1.5 min; 55°C for 1 min; 72°C for 1.5 min, and the reaction mixture as consequently incubated at 95°C for 1.5 min; at 55°C for 1 min; and at 72°C for 5 min. Ten of the PCR product was subjected to an electrophoresis on an 1.0% agarose gel at 100 volts for 30 min. The gel was stained in EtBr solution for 10 min, washed with distilled water and photographed.

*IL-6 CAT assay:* The plasmid IL-6-CAT was constructed by fusing IL-6 promoter (from −1180 to +13) and the bacterial CAT transcription unit.[31] On the day before DNA transfection, HeLa cells were plated at a density of 5 × $10^5$ cells per 60 mm Petri dishes in DMEM supplemented with 5% FBS. On day 2, 3 h before transfection, fresh DMEM containing 1% FBS was added and cells were transfected by the calcium phosphate-DNA co-precipitation method with a total of 8 µg of IL-6-CAT DNA. After 18 h, cells were washed with serum-free medium and added with fresh DMEM containing 1% FBS. Then cells were further incubated for 18 h in the presence of 10 µg/ml of LPS plus 100 ng/ml of PMA and/or 10 µg/ml of EXT.B. The cell extracts were heat-inactivated at 65°C for 10 min and centrifuged and assayed at 37°C for 2 h with the equal amounts of protein (20 µg) as measured by using Bio-Rad protein assay kit. The CAT expression was detected by TLC and autoradiography.

*Collagen synthesis:* Collagen released from the fibroblast cells was measured by indirect ELISA using anti-type I collagen antibody (Southern Biotechnology). Collagen synthesized in the lung tissue of the rats was assayed by measuring the amount of hydroxy proline.[32] To measure the amount of synthesized collagen by rat pulmonary fibroblasts, collagen (Sigma, type I) as an internal control group was dissolved thoroughly in 1 M acetic acid containing 1 mg/ml of pepsin, and the solution was serially diluted 5-fold with coating buffer (0.05 M carbonate, pH 9.6) in a concentration ranging from 1 μg to 16 pg. The diluted solutions were added to the wells of a flat-bottomed microtiter plate (Dynatech, Cantilly, VA, USA, Immulon 2) in the amount of 100 μl/well. In addition, 1 ml of the culture supernatants of rat pulmonary fibroblast were concentrated up to 10- to 20-fold using a speedvac dryer (Savant, Hicksville, NY, USA) and dissolved in 100 μl of coating buffer (0.1 M $NaHCO_3$, 0.02% $NaN_3$; pH was adjusted to 9.6 with $Na_2CO_3$), and the solution was added to the wells in an amount of 100 μl/well and then incubated at 4°C overnight. The plate was washed three times with washing buffer (PBS, 0.05% Tween 20, pH 7.4), and 2% bovine serum albumin (BSA, Sigma) was added to the wells in an amount of 100 μl/well. The plate was incubated at room temperature for 2 h to block the uncoated parts. The plate was washed four times with the same buffer as above, and alkaline phosphatase-conjugated rabbit anti-goat IgG (Cappel, Dunham, NC, USA) which was 1 000-fold diluted with a dilution buffer (0.05 M Tris-HCl, 1 mM $MgCl_2{\cdot}6H_2O$, 0.15 M NaCl, 0.02% $NaN_3$, 1% BSA, 0.05% Tween 20, pH 8.1) was added to the wells in an amount of 100 μl/well. The plate was incubated at 37°C for 2 h and then washed three times with the same buffer. To the wells was added 100 μl/well of *p*-nitrophenyl phosphate which was diluted with substrate buffer (0.05 M $NaHCO_3$, 10 mM $MgCl_2{\cdot}6H_2O$, pH 9.8) in a concentration of 1 mg/ml, and the O.D. was determined using an ELISA reader at 405 nm. The amount of produced collagen was calculated from the O.D. value with reference to that of the internal control group.

*Production of reactive oxygen species:* The amount of $H_2O_2$ was determined by a microassay employing a 96-well microplate as follows. $5 \times 10^5$ cells of human monocytes/macrophages were added to each well containing RPMI 1640 medium, and 25 μl of horseradish peroxidase (500 μg/ml; type II, Sigma) and 75 μl of phenol red (1 mg/ml) were added to each of the wells. Thereafter, the cells were treated with 10 μg/ml of Extract A for 1 h, stimulated with 100 μg/ml silica and then reacted at 37°C for 60 min. When the incubation was completed, 3 M NaOH was added to the wells in an amount of 25 μl/well to stop the reaction and O.D. was measured at 620 nm by using an ELISA reader. The amount of $H_2O_2$ was determined by employing a standard curve prepared using the diluted standard $H_2O_2$ (Sigma).

For the purpose of measuring the amount of produced $O_2^-$, monocytes/macrophages suspended in RPMI 1640 medium in a concentration of $1 \times 10^6$ cells/800 μl was added to the wells of a 24-well plate, and 10 μg/ml of superoxide dismutase (SOD, Sigma) was added to the empty wells. The plate was stored at 37°C for 2 min, and cytochrome C (3 mg/ml, Sigma) was added to the wells in a concentration of 100 μl/well. The cells were treated with 10 μg/ml of Extract A for 1 h, and stimulated at 37°C for 20 min by adding 100 μg/ml silica. The reaction was terminated by adding 1 mM *N*-ethylmaleimide (Sigma) to the wells and the culture was centrifuged at 1 600 × ***g*** for 10 min to obtain the supernatants. The change of color caused by the reduction of cytochrome C was measured at 550 nm using an UV-visible spectrophotometer (Kontron Instrument, Milan, Italy). The amount of produced $O_2^-$ was represented by the concentration of SOD which can suppress the reduction of cytochrome C in $1 \times 10^6$ cells for 20 min, by employing the extinction coefficient of cytochrome C ($E_{550nm} = 1.83 \times 10^4\ mM^{-1}\ cm^{-1}$).

*Experimental silicosis and cirrhosis model:* To induce the experimental hepatocirrhosis in 4-week aged male Sprague-Dawley rats,[33] 0.1 ml/100 g of body weight of $CCl_4$ solution (50% $CCl_4$ + 50% corn oil) was injected intraperitoneally to the rats twice a week, and 0.2 ml each of Extract A, B, C or D was administered orally at the time of the injections of $CCl_4$ twice a week. After 13 weeks, each of the rats was anaesthetized with ether and blood samples were obtained from the heart to determine the levels of serum glutamic-oxaloacetic transaminase (sGOT) and serum glutamic-pyruvic transaminase (sGPT).

For the pathohistological examination of the livers separated from the rats, the liver was fixed in 10% aqueous solution of neutral formalin and then embedded in paraffin. The embedded tissue was sectioned into 5 mm slices, stained with haematoxylin eosin and Masson's trichrome, and then observed under the microscope.

### Results

*Cytotoxicity of extracts of* S. tetrandra *S. Moore:* The cytotoxicity of the extracts of *S. tetrandra* S. Moore were determined as described Materials and Methods. Figure 1 shows the relative values of optical density of the sample with respect to the concentration of Extract A or B when the optical density of the control group which was not treated with Extract A or B is regarded as

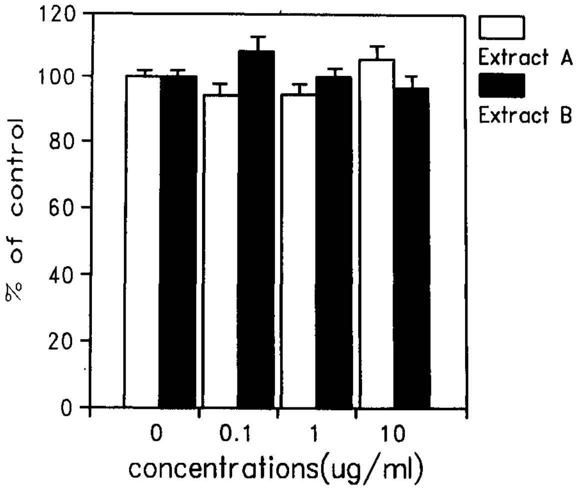

FIG. 1. Cytotoxicity of *S. tetrandra* S. Moore on human monocytes/macrophages. Human monocytes/macrophages (1 × $10^5$ cells/well) were isolated and incubated with various concentrations of *S. tetrandra* S. Moore (Extract A or B) for 48 h at 37°C. Then the cells were further incubated with 0.5 mg/ml of 3-4,5-dimethylthiazol-2,5-diphenyltetrazolium bromide (MTT) for 4 h. The cells were pelleted and resuspended in 100 μl of acidified prophylalcohol. The samples were mixed with 20 μl of 3% SDS and read on an ELISA reader at 540 nm. Data represent the standard deviation (SD) of five different determinants.

100%. The samples treated with Extract A show no difference from the control group until the concentration of Extract A reaches 10 μg/ml, and the samples treated with Extract B show similar results. Therefore, it indicated that Extracts A and B had no cytotoxicity at concentrations lower than 10 μg/ml, therefore, hereinafter, all the tests were carried out within this concentration. Both Extracts A and B showed cytotoxicity at the concentration of 100 μg/ml. *In vivo* oral treatment of Extracts C and D showed no apparent toxicity to the rats (death, loss of weight, etc.) when it was administered twice a week for 17 weeks (data not shown).

*Inhibition of IL-6 production by extracts of* S. tetrandra *S. Moore:* Human monocytes/macrophages were incubated with 0.1 to 10 μg/ml of Extract A or B for 1 h and treated with 100 μg/ml of silica for 48 h. The culture was centrifuged to obtain the culture supernatants, which were then dialysed against PBS. IL-6 production was measured by IL-6 bioassay using B9.55 cell line. Figure 2A shows the relative values of the

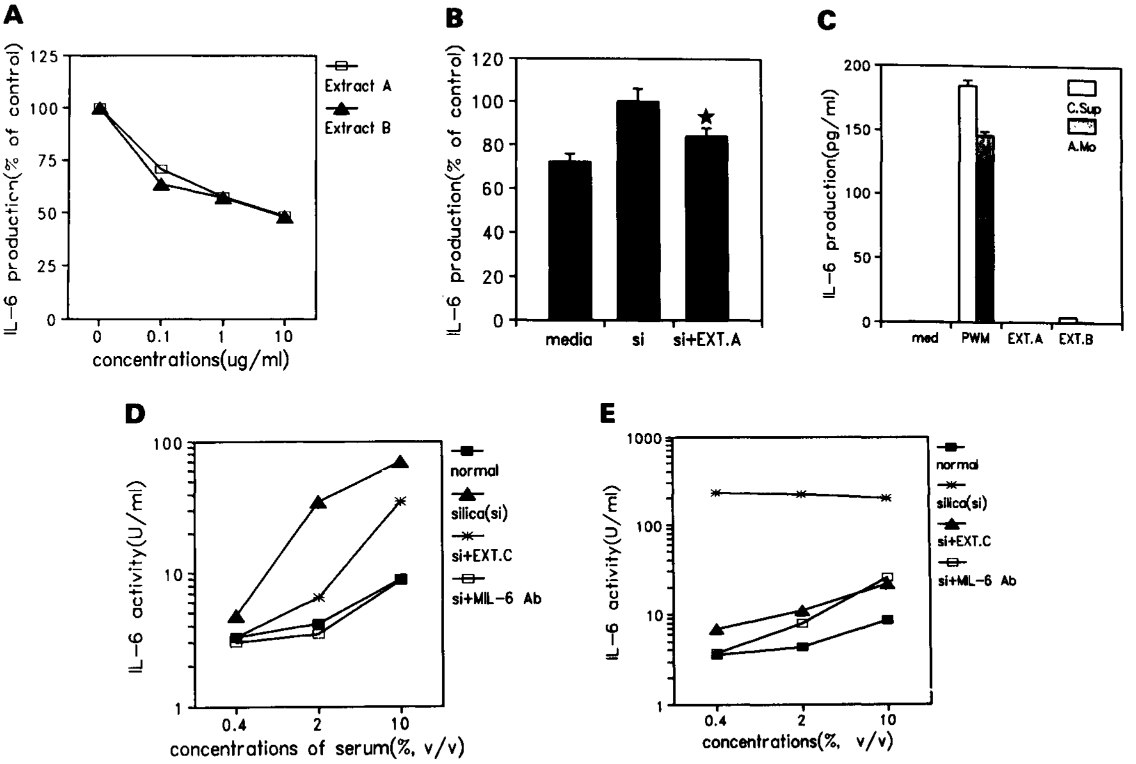

FIG. 2. Inhibitory effects of extracts of *S. tetrandra* S. Moore on IL-6 production. (A) Human monocytes/macrophages (1 × $10^5$/well) were incubated with various concentrations of *S. tetrandra* S. Moore for 1 h and stimulated with 100 μg/ml silica. After 48 h incubation, the culture supernatants were collected, centrifuged, dialysed, and filtered to remove the extracts. IL-6 production was bioassayed as described in Materials and Methods (control, silica only; 32 U/ml IL-6). (B) Rat alveolar macrophages (1 × $10^6$ cells/well) were incubated with 10 μg/ml *S. tetrandra* S. Moore for 1 h, stimulated with silica, and assayed for IL-6 as described in (A) (control, silica only; 28 U/ml). Data show the mean ± SD of three different determinants (★, $p < 0.05$). (C) The culture supernatants of human monocytes/macrophages (C. Sup) or of rat alveolar macrophages (A. Mo) treated with 10 μg/ml PWM and 10 μg/ml extracts were analysed for IL-6 concentration using IL-6 ELISA. (D) The experimental silicosis was induced as described in Materials and Methods. Then, Extract C (40 mg/injection) was administered orally, or murine IL-6 polyclonal antibody (250 μg/injection) was injected intravenously, into the silicosis rats twice per week for 17 weeks. IL-6 activities in the sera were determined as described in Materials and Methods. (E) The culture supernatants of pulmonary fibroblasts isolated from silicosis rats (D) were analysed for IL-6 activity.

amount of incorporated [$^3$H]thymidine with respect to the concentration of Extract A or B compared with those of the control group. IL-6 production by monocyte/macrophage was inhibited by Extract A or B in a dose-dependent mode, and it was inhibited by 50% with 10 μg/ml of Extract A or B. As shown in Fig. 2B, it was observed that the IL-6 production by rat alveolar macrophages was also inhibited by Extract A. As blank controls for possible residual effects of compounds and silica on bioassay, the same concentrations of compounds and silica were mixed with fresh media and dialysed as the culture supernatants were prepared. These blank controls had no effects on bioassay (data not shown). The results of IL-6 bioassay were further confirmed by IL-6 ELISA (Fig. 2C). In this case, cells were treated with 10 μg/ml pokeweed mitogen (PWM) and 10 μg/ml extracts. Extract A and B showed dramatic inhibitory effects on IL-6 production. Next, the effects of *S. tetrandra* S. Moore on IL-6 production *in vivo* were analysed. In the experimental silicosis model, IL-6 activities in the serum (Fig. 2D) and in the culture supernatants of pulmonary fibroblasts isolated from the rats treated with compounds or anti-murine IL-6 monoclonal antibody (Fig. 2E) were determined as described in Materials and Methods. IL-6 activity was inhibited by injecting anti-IL-6 antibody as expected. And its activity was also inhibited by Extract C in both cases, indicating that the extracts of *S. tetrandra* S. Moore exhibited the inhibitory effects on IL-6 production in the animal silicosis models.

*Suppression of IL-6 gene expression by extracts of* S. tetrandra *S. Moore:* In addition, to confirm the effects of the extracts of *S. tetrandra* S. Moore on the IL-6 gene expression, the effect of Extract B on synoviocytes which were obtained from the patients with rheumatoid arthritis caused by the overproduction of IL-6[18] was analysed. As can be seen in Fig. 3A, the expression of constantly expressed adenine phosphoribosyl transferase (APRT) RNA was not influenced by Extract B, while IL-6 RNA expression was significantly inhibited by 10 μg/ml of the Extract B. When IL-6-CAT construct[31] was used to see the effects of Extract B on IL-6 promoter activity, the IL-6 promoter activity induced by LPS plus PMA was suppressed about 60% by 10 μg/ml of the Extract B. However, acanthoic acid (A), another natural product which had no effect on IL-6 production,[34] did not inhibit the IL-6 expression (Fig. 3B,C). These results indicated that the extracts of *S. tetrandra* S. Moore suppressed IL-6 production at the transcriptional level, and further studies are required to know the exact

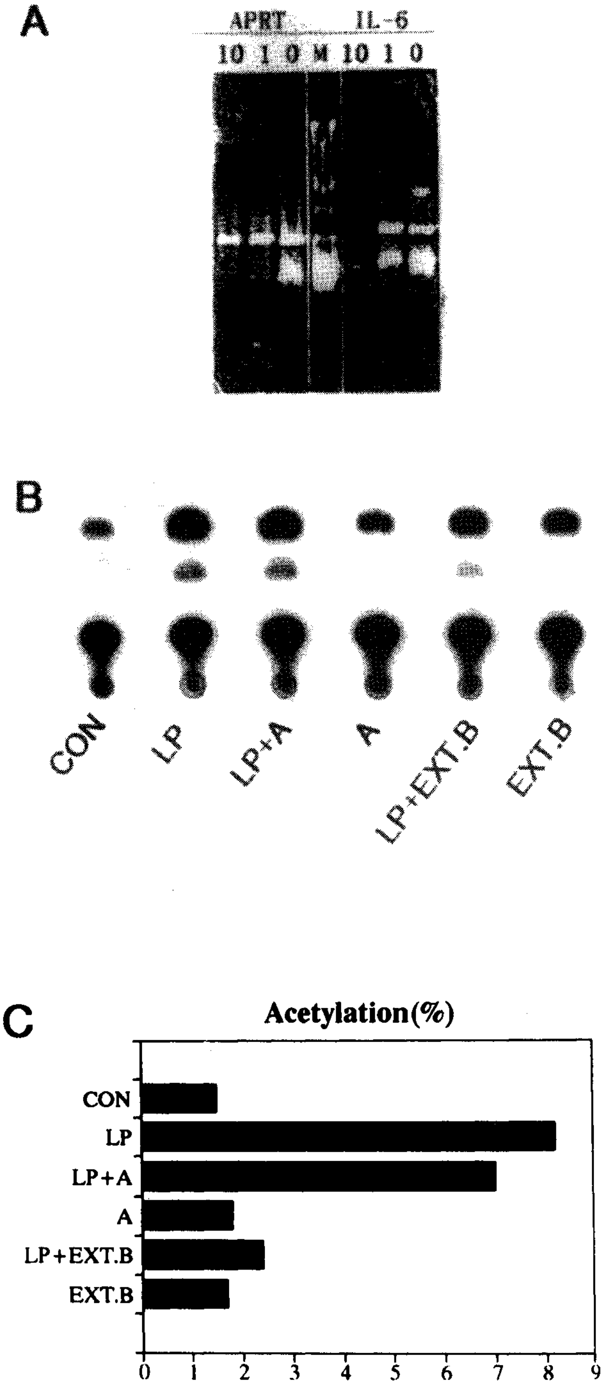

FIG. 3. Suppression of IL-6 gene expression by Extract B. (A) 1 μg/ml or 10 μg/ml of Extract B was added to the synoviocytes of patients with rheumatoid arthritis (1.5 × $10^6$ cells/well) and was incubated at 37°C, 5% $CO_2$ for 1 h and treated with 100 μg/ml silica for 12 h. RNA were prepared, and IL-6 or APRT gene expression was measured by RT-PCR using specific primers as described in Materials and Methods. (B) 8 μg of IL-6-CAT construct was transfected into HeLa cells by the calcium phosphate-DNA coprecipitation method. The cells were incubated in DMEM containing 1% FBS for 18 h. Cells were then treated with 10 μg/ml LPS plus 100 ng/ml PMA (LP), 10 μg/ml EXT.B, or 10 μg/ml acanthoic acid (A) for 18 h. The CAT expression was measured as described in Materials and Methods. (C) The CAT activity was calculated as follows: percentage acetylated = (cpm in acetylated species)/(cpm in acetylated species + cpm in non-acetylated chloramphenicol).

suppressive mechanism of *S. tetrandra* S. Moore on IL-6 gene expression.

*Inhibition of silicosis by extracts of* S. tetrandra *S. Moore:* IL-6 is known as a cytokine which causes fibrogenesis and induces collagen synthesis in rat fibroblasts.[35] Based on the observation of the inhibitory effects on the IL-6 production, their inhibitory effect on the collagen synthesis in rat pulmonary fibroblasts and pulmonary tissues was determined. As a result, it was observed that the amount of synthesized collagen was significantly decreased in the culture supernatants of rat pulmonary fibroblasts which were treated with 10 µg/ml of Extract A compared with the control (Fig. 4A). Furthermore, *in vivo* effects of *S. tetrandra* S. Moore (Extract C) on the collagen synthesis in rat pulmonary tissues was tested by injecting 500 mg of silica and 40 mg of Extract C orally into the rats twice a week for 17 weeks, and then the amount of hydroxyproline was measured as described in Materials and Methods. In Fig. 4B, the amount of collagen synthesized by rat pulmonary tissues from the rats treated with silica only (si) or treated with silica and dimethylsulphoxide (si + DMSO) increased about twice compared with the control, while the amount of synthesized collagen decreased by 50% in rat pulmonary tissue treated with silica, DMSO and Extract C (si + EXT.C). These results showed that the extracts of *S. tetrandra* S. Moore had antifibrogenic activity *in vitro* and *in vivo*.

Inflammatory responses are known as a cascade reaction comprising the secretion of various cytokines such as IL-1, IL-6, and TNF-α

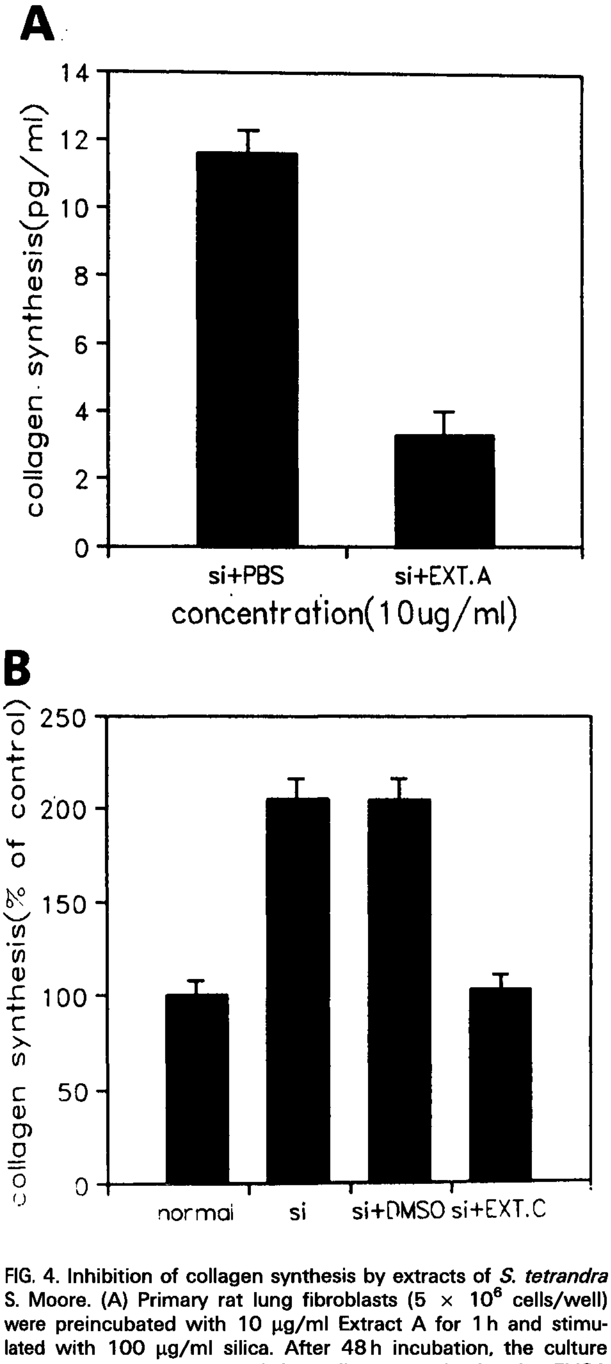

FIG. 4. Inhibition of collagen synthesis by extracts of *S. tetrandra* S. Moore. (A) Primary rat lung fibroblasts (5 × $10^6$ cells/well) were preincubated with 10 µg/ml Extract A for 1 h and stimulated with 100 µg/ml silica. After 48 h incubation, the culture supernatants were assayed for collagen production by ELISA using anti-collagen type I antibody. (B) The experimental silicosis was induced as described in Materials and Methods. Then, Extract C (40 mg/injection) was orally injected into the silicosis rat twice per week for 17 weeks. The lung tissue (0.1 g) was hydrolyzed and assayed for collagen content by measuring hydroxy proline. Data represent the percentages of control (normal lung).

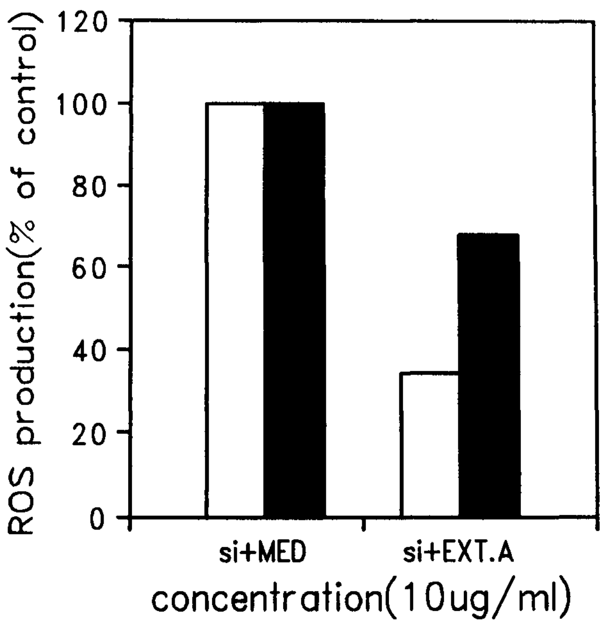

FIG. 5. Inhibition of production of reactive oxygen species by extract of *S. tetrandra* S. Moore. For hydrogen peroxide analysis, human monocytes/macrophages (5 × $10^5$ cells/ml) pretreated with 10 µg/ml Extract A for 1 h were stimulated with 100 µg/ml silica for 1 h in the presence of horseradish peroxidase and phenol red. For superoxide anion analysis, human monocytes/macrophages (1 × $10^6$ cells/800 µl) pretreated with 10 µg/ml Extract A were stimulated with 100 µg/ml silica for 20 min in the presence of cytochrome C. □, superoxide anion; ■, hydrogen peroxide.

from immune cells stimulated by various stimulants; production of phospholipase $A_2$, lysosomal enzyme, reactive oxygen species, etc. by other immune cells stimulated by various cytokines; and damage of tissues induced by the inflammatory mediators.[36] The ability of the extracts of *S. tetrandra* S. Moore to block the inflammatory reactions was tested by measuring their inhibitory activity to the production of reactive oxygen species such as $H_2O_2$ and $O_2^-$. As can be seen from Fig. 5, the amounts of $H_2O_2$ and $O_2^-$ decreased significantly in the silica-stimulated and Extract A-treated monocytes/macrophage (si + EXT.A) in contrast with the control group treated with only silica (si + MED).

*Inhibition of hepatocirrhosis by extracts of* S. tetrandra *S. Moore:* Cirrhosis is another fibrotic disease in the liver. Anti-inflammatory effects of *S. tetrandra* S. Moore were analysed in the experimental cirrhosis model. When compared with the blood sample obtained from the rats treated with $CCl_4$, DMSO, and PBS, sGOT values of the blood samples obtained from the rats treated with Extract A or C did not decrease, but those of samples obtained from the rat treated with Extract D or B decreased by 20% and 40%, respectively (Fig. 6A). Further, sGPT in the blood samples obtained from the rats treated with Extract B decreased more than 60% (Fig. 6B).

In the liver of the rats administered with $CCl_4$ only (Fig. 7B), the nodule formation of hepatic lobules with the thickened fibrous bands was remarkable compared with the normal liver (Fig. 7A). In the liver of the rats administered with $CCl_4$ and Extract B (Fig. 7D), the fibrous bands surrounding the nodule of hepatic lobule were thinner than those of the liver obtained from the rats treated with $CCl_4$ only, and many nodules were incomplete. Also, the regenerative change of hepatic cells decreased compared with that of the liver obtained from the rats treated with $CCl_4$ only. In the liver of the rats administered with $CCl_4$ and Extract A (Fig. 7C), the suppressive effect on the hepatocirrhosis was not dominant as seen in the case of Extract B.

### Discussion

Inflammation is a localized or systemic response induced by infection or injury to tissues. Right after exposure to injurious agents, monocytes and macrophages synthesize and release a variety of inflammatory mediators such as oxygen free radicals, proteases, platelet-activating factors, and proinflammatory cytokines.[1,2] They are major inflammatory mediators that modulate fibroblast proliferation, neutrophil function, and collagen synthesis. Once macrophages infiltrated into inflammatory sites, NADPH-oxidase in the membrane is activated to produce the oxygens. The oxygens are then reduced to superoxide anion by receiving an electron from NADPH, and then dismutated to hydrogen peroxide. These reaction oxygens then damage the tissues in the inflammatory sites.

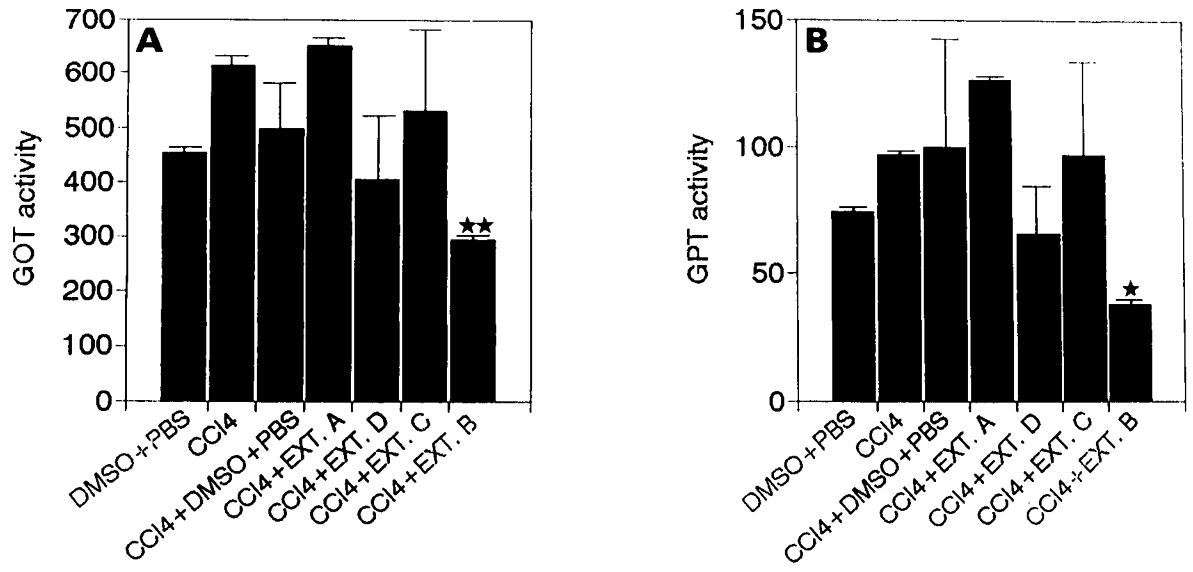

FIG. 6. Effects of Extracts of *S. tetrandra* S. Moore on serum GOT and GPT of cirrhotic rats. The experimental cirrhosis was induced by $CCl_4$ as described in Materials and Methods. Then, 40 mg each of Extract A, B, C, D was orally injected into the cirrhotic rats for 13 weeks. The sera were obtained and analyzed for GOT and GPT (★★, $p < 0.01$; ★, $p < 0.05$).

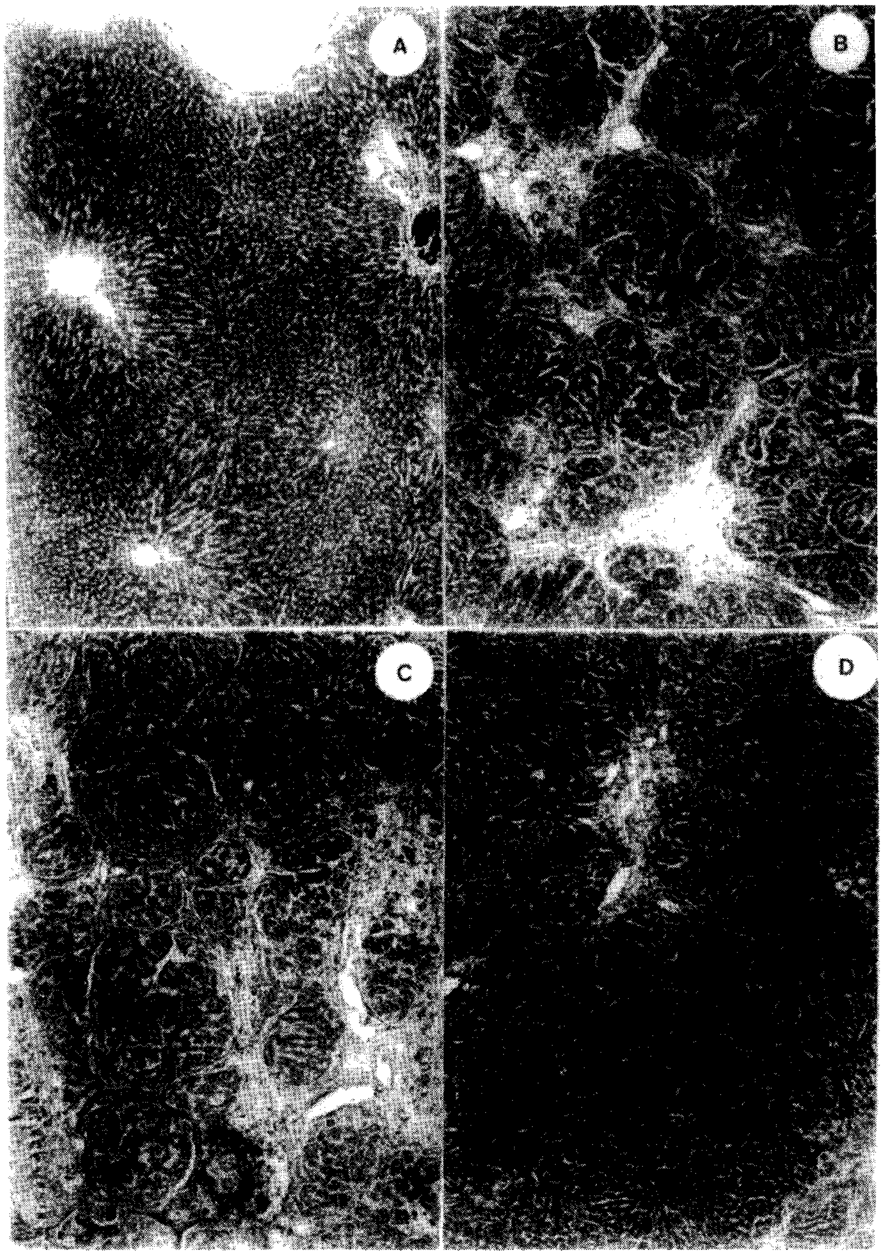

FIG. 7. Extracts of *S. tetrandra* S. Moore suppressed the experimental cirrhosis. 40 mg each of Extract A, B was injected into cirrhotic rats for 13 weeks. The liver was fixed, stained with Masson's trichrome stain for histological analysis. (A) normal liver, (B) $CCl_4$, (C) $CCl_4$ and Extract A, (D) $CCl_4$ and Extract B. Data shown the mean $\pm$ SD of three different determinants.

Therefore, an excessive quantity of these cytokines and free radicals caused tissue destruction in the inflammatory sites and they are involved in many immune diseases and inflammatory diseases. To inhibit their action, several protein antagonists such as IL-1ra, monoclonal antibodies to proinflammatory cytokines, and these cytokine receptors have been developed to block acute and chronic inflammation. But these protein antagonists have some limitations such as stability and delivery to be used clinically as anti-inflammatory agents.

Two major aspects of the proliferation of fibroblasts and the production of collagen are the hallmarks of fibrosis. IL-6 has been known to be involved in this process in connection with other fibrogenic cytokines. To modulate IL-6 production, *S. tetrandra* S. Moore, known as anti-inflammatory agent, was tested in different inflammatory systems. First, several aspects of IL-6 in chronic inflammation including silicosis, rheumatoid arthritis, and liver cirrhosis were evaluated. Significantly elevated levels of IL-6 activity were observed in silica-treated monocytes/macrophages, and in the sera of $CCl_4$-treated rats and the patients with rheumatoid arthritis, indicating that IL-6 is a key element in an inflammatory cytokine network. The culture supernatants of human monocytes/macrophages treated with *S. tetrandra* S. Moore contained far less IL-6 activity compared with those treated with silica alone. In addition, it suppressed the IL-6 gene expression, collagen synthesis, and the production of reactive oxygen from several inflammatory cells, suggesting that it had potent anti-fibrogenic effects. But it cannot be ruled out that *S. tetrandra* S. Moore may regulate the production of other proinflammatory cytokines. Actually, Extract B of *S. tetrandra* S. Moore showed the inhibitory effects on IL-1 production (29.1% and 45% inhibition at the concentration of 1 μg/ml and 10 μg/ml, respectively) and on TNF-α production (5.2% and 18.6% inhibition at the concentration of 1 μg/ml and 10 μg/ml, respectively). The further fractionation of the extracts to identify the effective components inhibiting each cytokine production is now in progress.

To evaluate the *in vivo* effects of *S. tetrandra* S. Moore, two different experimental models, in which IL-6 is known to be involved, were tested. Silicosis is a chronic lung inflammation. By the exposure to stimuli, alveolar macrophages are activated and secrete many fibrogenic mediators. *S. tetrandra* S. Moore strongly suppressed the experimental silicosis induced by silica treatment. It reduced the collagen production, and granuloma formation and fibrosis in the lung.

Hepatocirrhosis is characterized by the fibrogenesis of the whole liver, complete disruption of liver parenchyma by the fibrous septa, and formation of regenerative nodules. It is derived mostly from a chronic hepatitis or chronic alcoholism, however, the precise causes are unknown. We previously reported that *in vivo* administration of IL-6 could induce the early stage of liver cirrhosis.[37] In a hepatocirrhosis patient, the amount of cytokines, e.g. IL-6 which is involved in the inflammation and fibrogenesis, is in an increased state. Based on its effects on IL-6 production, these inhibitory effects *in vivo* might be due to reduction of IL-6 and/or other inflammatory mediators induced by IL-6 such as reactive oxygens free radicals.

Various root extracts of *S. tetrandra* S. Moore which inhibit the production of interleukin-6, can be used for the preparation thereof and pharmaceutical compositions comprising extracts which are effective for the treatment of immune diseases caused by the overproduction of interleukin-6. Each of Extracts A, B, C and D exhibited an anti-inflammatory effect, inhibited the synthesis of collagen and the production of the reactive oxygen species and reduced GOT and GPT level in the serum. Therefore, they can be employed alone or in combination with each other in a pharmaceutical composition for the treatment of such immune diseases caused by an excessive production of IL-6 as rheumatoid arthritis, hepatocirrhosis, psoriasis, multiple myeloma, cardiac myxoma, and silicosis. These extracts seemed to have the similar effects on IL-6 production, but they showed some different effects on experimental animal models. The Extract B was most effective in suppressing serum GOT and GPT level, and hepatic cirrhosis. Preferably the Extract B may be applied for the treatment of inflammatory disease, arthritis and autoimmune hepatocirrhosis, but the subsequent studies are required to identify the active compounds as mentioned above.

In summary, significantly elevated levels of IL-6 activity and concentration were observed in silica-treated human monocytes/macrophages, $CCl_4$-or silica-treated rats, and the patients with rheumatoid arthritis. In addition, IL-6 itself augmented fibrogenic events such as fibroblast or synoviocyte proliferation, and collagen synthesis. *S. tetrandra* S. Moore possesses the ability to inhibit the production of IL-6 and inflammatory mediators in a concentration-dependent fashion. It was also effective in experimental silicosis and cirrhosis models. Considering its effects on cytokine regulation and *in vivo* effects, it can be used as an anti-fibrosis agent in the treatment of many inflammatory diseases. Further biological and immunological analysis of its isolated compounds can lead to the development of more specific anti-inflammatory therapeutics for the treatment of various immune diseases caused by an excessive production of IL-6 and other inflammatory mediators.

### References

1. Kovacs EJ. Fibrogenic cytokines; the role of immune mediators in the development of scar tissue. *Immunol Today* 1991; **12:** 17–23.
2. Wahl SM, Wahl LM. Modulation of fibroblast growth and function by monokines and lymphokines. *Lymphokines* 1981; **2:** 179–200.
3. Shalaby MR, Aggarwal BB, Rinderknecht E, Svedersky LP, Finkle BS, Palladino MA. Activation of human polymorphonuclear neutrophil functions by interferon-γ and tumor necrosis factors. *J Immunol* 1985; **135:** 2069–2073.
4. Schmidt JJ, Mizel SS, Cohen D, Green I. Interleukin-1, a potential regulator of fibroblast proliferation. *J Immunol* 1982; **128:** 2177–2182.
5. Davis GS. pathogenesis of silicosis; current concepts and hypothesis. *Lung* 1986; **164:** 139–154.
6. Kovacs EJ. Fibrogenic cytokines; the roles of immune mediators in the development of scar tissue. *Immunol Today* 1991; **12:** 17–23.
7. Kumar RK. Quantitative immunohistologie assessment of lymphocyte populations in the pulmonary inflammatory response to intratracheal silica. *Am J Pathol* 1989; **135:** 605–614.
8. Castilla A, Prieto J, Fausto N. Transforming growth factor-β1 and α in chronic liver disease. *N. Engl J Med* 1991; **324:** 933–940.
9. Czaja MJ, Weiner FR, Flander KC, Giambrone MA, Wind R, Biempica L, Zern MA. *In vitro* and *in vivo* association of transforming growth factor-β1 with hepatic fibrosis. *J Cell Biol* 1989; **108:** 2477–2482.
10. Hirano T, Akira S, Taga T, Kishimoto T. Biological and clinical aspects of interleukin-6. *Immunol Today* 1990; **11:** 443–449.

11. Muraguchi A, Kishimoto T, Miki Y, Kuritani T, Kaieda T, Yoshizaki K, Yamamura Y. T cell-replacing factor (TRF) induced IgG secretion in a human B blastoid cell line and demonstration of acceptors for TRF. *J Immunol* 1991; **127:** 412–416.
12. Toshio H, Kiyoshi Y, Hisashi H, Tetsuya T, Yasuo W, Tadashi M, Shinichiro K, Koichi N, Koichi K, Akihiro I, Susumu T, Fumio S, Hiroshi M, Yoshiyuki T, Tadatsugu T, Tadamitsu K. Complementary DNA for a novel human interleukin (BSF-2) that induces B lymphocytes to produce immunoglobulin. *Nature* 1986; **324:** 73–76.
13. Snick VJ, Cayphas S, Yink A, Uyttenhove C, Coulie PG, Rubira MR, Simpson RJ. Purification and $NH_2$-terminal amino acid sequence of a T-cell-derived lymphokine with growth factor activity for B-cell hybridomas. *Proc Natl Acad Sci USA* 1986; **83:** 9679–9683.
14. Koike K, Nakahata T, Takagi M, Kobayashi T, Ishigaro A, Tsuji K, Naganuma K, Okano A, Akiyama Y, Akabane T. Synergism of BSF-2/interleukin-6 and interleukin-3 on development of multipotential hemopoietic progenitors in serum-free culture. *J Exp Med* 1988; **168:** 879–890.
15. Lotz M, Jirik F, Kobouridis P, Tsoukas C, Hirano T, Kishimoto T, Carson DA. B cell stimulatory factor 2/interleukin-6 is a costimulant for human thymocytes and T lymphocytes. *J Exp Med* 1988; **167:** 1253–1258.
16. Geiger T, Andus T, Klapproth J, Hirano T, Kishimoto T, Heinrich PC. Induction of rat acute-phase proteins by interleukin-6 *in vivo*. *Eur J Immunol* 1988; **18:** 717–721.
17. Satoh T, Nakamura S, Taga T, Matsuda T, Hirano T, Kishimoto T, Kaziro Y. Induction of neuronal differentiation in PC12 cells by B cell stimulatory factor 2/ interleukin-6. *Mol Cell Biol* 1988; **8:** 3546–3549.
18. Hirano T, Matsuda T, Tuner M, Miyasaka N, Buchan G, Tang B, Sato K, Shimizu M, Maini R, Feldmann M, Kishimoto T. Excessive production of interleukin-6/B cell stimulatory factor-2 in rheumatoid arthritis. *Eur J Immun* 1988; **18:** 1797–1801.
19. Diviere J, Content J, Denys C, Vandenbussche P, Schandene L, Wybran J, Dupont E. High interleukin-6 serum levels and increased production by leukocytes in alcoholic liver cirrhosis. Correlations with IgA serum levels and lymphokine production. *Clin Exp Immunol* 1989; **77:** 221–225.
20. Grossman RM, Krueger J, Yojurish D, Granelli-Piperno A, Murphy DP, May LT, Kupper TS, Sehgal PB, Gottlieb AB. Interleukin-6 is expressed in high levels in psoriatic skin and stimulates proliferation of cultured human keratinocytes. *Proc Natl Acad Sci USA* 1989; **86:** 6367–6371.
21. Bataille R, Jourdan M, Zhang XG, Kein B. Serum levels of interleukin-6, a potent myeloma cell growth factor, as a reflect of disease severity in plasma cell dyscrasias. *J Clin Invest* 1989; **84:** 2008–2011.
22. Jourdan M, Bataille R, Seguin J, Zhang XG, Chaptal PA, Klein B. Constitutive production of interleukin-6 and immunologic features in cardiac myxomas. *Arthritis Rheum* 1990; **33:** 398–402.
23. Miles SA, Rezai AR, Salazar-Gonzalez JF, Vander-Meyden M, Stevens RH, Logan DM, Mitsuyasu RT, Taga T, Hirano T, Kishimoto T, Martinez-Maza O. AIDS kaposi sarcoma-derived cells produce and respond to interleukin-6. *Proc Natl Acad Sci USA* 1990; **87:** 4068–4072.
24. Suzuki H, Yasukawa K, Saito T, Goitsuka R, Hasegawa A, Ohsugi Y, Taga T, Kishimoto T. Anti-human interleukin-6 receptor antibody inhibits human myeloma growth *in vivo*. *Eur J Immunol* 1991; **22:** 1989–1993.
25. Seow WK, Ferrante A, Goh DB, Chalmers AH, Li SY, Thong YH. *In vitro* immuno suppressive properties of the plant alkaloid tetrandrine. *Int Arch Allergy Appl Immunol* 1988; **85:** 410–415.
26. Seow WK, Ferrante A, Si-ying L, Thong YH. Antiphagocytic and antioxidant properties of plant alkaloid tetrandrine. *Int Arch Allergy Appl Immunol* 1988; **85:** 404–409.
27. Seow WK, Ferrante A, Si-Ying L, Thong YH. Suppression of human monocyte interleukin 1 production by the plant alkaloid tetrandrine. *Clin Exp Immunol* 1989; **75:** 47–51.
28. Ferrante A, Seow WK, Rowan-Kelly B, Thong YH. Tetrandrine, a plant alkaloid, inhibits the production of tumor necrosis factor-alpha (cachectin) by human monocytes. *Clin Exp Immunol* 1990; **80:** 232–235.
29. Castranova V, Kang JH, Moore MD, Pailes WH, Frazer DJ, Schwegler-Berry D. Inhibition of stimulant-induced activation of phagocytic cells with tetrandrine. *J Leuk Biol* 1991; **50:** 412–422.
30. Alley MC, Scudiero DA, Monks A, Hursey ML, Czerwinski MJ, Fine DL, Abbott BJ, Mayo JG, Shoemaker RH, Boyd MR. Feasibility of drug screening with panels of human tumor cell lines using a microculture tetrazolium assay. *Cancer Res* 1988; **48:** 589–601.
31. Ray A, Tatter SB, May LT, Sehgal PB. Activation of the human '$\beta_2$-interferon/hepatocyte-stimulating factor/interleukin-6' promoter by cytokines, virus, and second messenger agonists. *Proc Natl Acad Sci USA* 1988, **85:** 6701–6705.
32. Huszar G, Maiocco J, Naftolin F. Monitoring of collagen and collagen fragments in chromatography of protein mixture. *Anal Biochem* 1980; **105:** 424–429.
33. Nakatsukasa H, Nagy P, Evarts RP, Hsia CC, Marsden E, Thorgeirsson SS. Cellular distribution of transforming growth factor-$\beta$1 and procollagen type I, III, and IV transcripts in carbon tetrachloride-induced rat liver fibrosis. *J Clin Invest* 1990; **85:** 1833–1843.
34. Kang HS, Kim YH, Lee CS, Lee JJ, Choi I, Pyun KH. Suppression of interleukin-1 and tumor necrosis factor-$\alpha$ production by acanthoic acid, (−)-pimara-9(11),15-dien-19-oic acid, and its antifibrotic effects *in vivo*. *Cell Immunol* 1996 (in press).
35. Kang HS, Choi I, Pyun KH. Effects of IL-6 and TNF-$\alpha$ in the pathogenesis of silicosis. *Kor J BRM* 1994; **4:** 97–109.
36. Pruzanski W, Vadas P. Phospholipase $A_2$—a mediator between proximal and distal effectors of inflammation. *Immunol Today* 1991; **12:** 143–146.
37. Choi I, Kang HS, Yang Y, Pyun KH. IL-6 induces hepatic inflammation and collagen synthesis *in vivo*. *Clin Exp Immunol* 1994; **95:** 530–535.

ACKNOWLEDGEMENT. This work was supported by the grants (HAN project HS020M and GG033S) from MOST, Republic of Korea.

**Received 28 May 1996; accepted 12 June 1996**

## References

[PDF_01024] Alley M. C., Scudiero D. A., Monks A., Hursey M. L., Czerwinski M. J., Fine D. L., Abbott B. J., Mayo J. G., Shoemaker R. H., Boyd M. R. (1988). Feasibility of drug screening with panels of human tumor cell lines using a microculture tetrazolium assay.. Cancer Res.

[PDF_00996] Bataille R., Jourdan M., Zhang X. G., Klein B. (1989). Serum levels of interleukin 6, a potent myeloma cell growth factor, as a reflect of disease severity in plasma cell dyscrasias.. J Clin Invest.

[PDF_01047] Choi I., Kang H. S., Yang Y., Pyun K. H. (1994). IL-6 induces hepatic inflammation and collagen synthesis in vivo.. Clin Exp Immunol.

[PDF_00942] Davis G. S. (1986). Pathogenesis of silicosis: current concepts and hypotheses.. Lung.

[PDF_00992] Grossman R. M., Krueger J., Yourish D., Granelli-Piperno A., Murphy D. P., May L. T., Kupper T. S., Sehgal P. B., Gottlieb A. B. (1989). Interleukin 6 is expressed in high levels in psoriatic skin and stimulates proliferation of cultured human keratinocytes.. Proc Natl Acad Sci U S A.

[PDF_00951] Hirano T., Akira S., Taga T., Kishimoto T. (1990). Biological and clinical aspects of interleukin 6.. Immunol Today.

[PDF_00984] Hirano T., Matsuda T., Turner M., Miyasaka N., Buchan G., Tang B., Sato K., Shimizu M., Maini R., Feldmann M. (1988). Excessive production of interleukin 6/B cell stimulatory factor-2 in rheumatoid arthritis.. Eur J Immunol.

[PDF_01032] Huszar G., Maiocco J., Naftolin F. (1980). Monitoring of collagen and collagen fragments in chromatography of protein mixtures.. Anal Biochem.

[PDF_00999] Jourdan M., Bataille R., Seguin J., Zhang X. G., Chaptal P. A., Klein B. (1990). Constitutive production of interleukin-6 and immunologic features in cardiac myxomas.. Arthritis Rheum.

[PDF_00971] Koike K., Nakahata T., Takagi M., Kobayashi T., Ishiguro A., Tsuji K., Naganuma K., Okano A., Akiyama Y., Akabane T. (1988). Synergism of BSF-2/interleukin 6 and interleukin 3 on development of multipotential hemopoietic progenitors in serum-free culture.. J Exp Med.

[PDF_00932] Kovacs E. J. (1991). Fibrogenic cytokines: the role of immune mediators in the development of scar tissue.. Immunol Today.

[PDF_00944] Kovacs E. J. (1991). Fibrogenic cytokines: the role of immune mediators in the development of scar tissue.. Immunol Today.

[PDF_00975] Lotz M., Jirik F., Kabouridis P., Tsoukas C., Hirano T., Kishimoto T., Carson D. A. (1988). B cell stimulating factor 2/interleukin 6 is a costimulant for human thymocytes and T lymphocytes.. J Exp Med.

[PDF_01002] Miles S. A., Rezai A. R., Salazar-González J. F., Vander Meyden M., Stevens R. H., Logan D. M., Mitsuyasu R. T., Taga T., Hirano T., Kishimoto T. (1990). AIDS Kaposi sarcoma-derived cells produce and respond to interleukin 6.. Proc Natl Acad Sci U S A.

[PDF_01035] Nakatsukasa H., Nagy P., Evarts R. P., Hsia C. C., Marsden E., Thorgeirsson S. S. (1990). Cellular distribution of transforming growth factor-beta 1 and procollagen types I, III, and IV transcripts in carbon tetrachloride-induced rat liver fibrosis.. J Clin Invest.

[PDF_01045] Pruzanski W., Vadas P. (1991). Phospholipase A2--a mediator between proximal and distal effectors of inflammation.. Immunol Today.

[PDF_01028] Ray A., Tatter S. B., May L. T., Sehgal P. B. (1988). Activation of the human "beta 2-interferon/hepatocyte-stimulating factor/interleukin 6" promoter by cytokines, viruses, and second messenger agonists.. Proc Natl Acad Sci U S A.

[PDF_00978] Satoh T., Nakamura S., Taga T., Matsuda T., Hirano T., Kishimoto T., Kaziro Y. (1988). Induction of neuronal differentiation in PC12 cells by B-cell stimulatory factor 2/interleukin 6.. Mol Cell Biol.

[PDF_00940] Schmidt J. A., Mizel S. B., Cohen D., Green I. (1982). Interleukin 1, a potential regulator of fibroblast proliferation.. J Immunol.

[PDF_01009] Seow W. K., Ferrante A., Goh D. B., Chalmers A. H., Li S. Y., Thong Y. H. (1988). In vitro immunosuppressive properties of the plant alkaloid tetrandrine.. Int Arch Allergy Appl Immunol.

[PDF_00936] Shalaby M. R., Aggarwal B. B., Rinderknecht E., Svedersky L. P., Finkle B. S., Palladino M. A. (1985). Activation of human polymorphonuclear neutrophil functions by interferon-gamma and tumor necrosis factors.. J Immunol.

[PDF_01006] Suzuki H., Yasukawa K., Saito T., Goitsuka R., Hasegawa A., Ohsugi Y., Taga T., Kishimoto T. (1992). Anti-human interleukin-6 receptor antibody inhibits human myeloma growth in vivo.. Eur J Immunol.

[PDF_00967] Van Snick J., Cayphas S., Vink A., Uyttenhove C., Coulie P. G., Rubira M. R., Simpson R. J. (1986). Purification and NH2-terminal amino acid sequence of a T-cell-derived lymphokine with growth factor activity for B-cell hybridomas.. Proc Natl Acad Sci U S A.

